# A Well-Balanced Unified Gas-Kinetic Scheme for Multicomponent Flows under External Force Field

**DOI:** 10.3390/e24081110

**Published:** 2022-08-12

**Authors:** Tianbai Xiao

**Affiliations:** Department of Mathematics, Karlsruhe Institute of Technology, 76131 Karlsruhe, Germany; tianbai.xiao@kit.edu

**Keywords:** fluid mechanics, kinetic theory, rarefied gas dynamics, multicomponent flows, well-balanced schemes

## Abstract

The study of the evolution of the atmosphere requires careful consideration of multicomponent gaseous flows under gravity. The gas dynamics under an external force field is usually associated with an intrinsic multiscale nature due to large particle density variation along the direction of force. A wonderfully diverse set of behaviors of fluids can be observed in different flow regimes. This poses a great challenge for numerical algorithms to accurately and efficiently capture the scale-dependent flow physics. In this paper, a well-balanced unified gas-kinetic scheme (UGKS) for a gas mixture is developed, which can be used for the study of cross-scale multicomponent flows under an external force field. The well-balanced scheme here indicates the capability of a numerical method to evolve a gravitational system under any initial condition to the hydrostatic equilibrium and to keep such a solution. Such a property is crucial for an accurate description of multicomponent gas evolution under an external force field, especially for long-term evolving systems such as galaxy formation. Based on the Boltzmann model equation for gas mixtures, the UGKS leverages the space–time integral solution to construct numerical flux functions and, thus, provides a self-conditioned mechanism to recover typical flow dynamics in various flow regimes. We prove the well-balanced property of the current scheme formally through theoretical analysis and numerical validations. New physical phenomena, including the decoupled transport of different gas components in the transition regime, are presented and studied.

## 1. Introduction

The challenge of modeling and simulating real gas evolution in engineering and environmental applications has attracted continuous attention from the CFD community. To be precise, the Earth’s atmosphere needs to be considered, at least as a binary mixture of nitrogen and oxygen under a gravitational field. Compared with the classical fluid dynamics of pure gas, theoretical and numerical studies on multicomponent gas systems under an external force field are very limited. The goal of this paper is to advance the cutting-edge research in this direction, with a particular focus on multiscale and non-equilibrium flows.

The characteristic scale and flow regime is usually categorized by the Knudsen number Kn. When Kn is large, the Boltzmann equation is established at the molecular mean free path and traveling time between successive intermolecular collisions. Such spatiotemporal scales can be referred to as the kinetic scale. Based on the first physical principle, it is natural to extend the Boltzmann equation to gas mixtures by tracking the evolution of each component. With a different molecular mass and gas constant *R*, different gas components transport with different velocity $u∼\sqrt{RT}$, where *T* is temperature, leading to strong non-equilibrium transport phenomena. Such an effect occurs dramatically when the mass ratio is large, such as the rounding motion of ions and electrons in plasma physics.

On the other hand, when Kn is small, the characteristic scale of flow structures is basically much larger than the mean free path, and a macroscopic model is favored to describe the flow evolution collectively. In the hydrodynamic limit, the Euler and Navier–Stokes equations are routinely used, where different gas components present consistent collective behavior. Additional constitutive equations are required to track the evolution of different components. Such additional equations can be the equations for the volume fraction, mass fraction, or ratio of specific heats of a mixture [1,2]. It is a nontrivial task since the information of particle interactions among different components at the kinetic scale is lost during the coarse-grained process and should be modeled back to the macroscopic system in a consistent fashion.

Different equations and the corresponding numerical algorithms are scale-dependent methods to describe flows at a certain level. However, in real-world gaseous flows, there may not exist a clear scale separation between different flow regimes. For example, under the gravitational field, the density varies significantly along the direction of force, as does the mean free path and local Knudsen number. As a result, the atmosphere can thus be divided into several layers, and a continuous variation of flow physics will emerge from the kinetic physics in the upper atmospheric layer to the hydrodynamics in the lower high-density region. Due to such an intrinsic multiscale nature, the corresponding numerical algorithm should have the capability of capturing the cross-scale flow physics effectively.

For a gas dynamic system under a steady external force field from an arbitrary initial condition, the entropy-increasing process leads to a hydrostatic equilibrium state. Such a static solution is achieved and preserved due to the balance between the external force and inhomogeneous fluxes. The capacity to capture such an equilibrium solution along a physically accurate path is the so-called well-balanced property, which is important for a numerical algorithm to solve long-term fluid dynamics under an external force field. For the equilibrium flow when Kn approaches zero, such as the gravitational Euler system, many efforts have been devoted to the construction of well-balanced schemes for single-component flow [3,4,5]. For more general gas dynamic equations with the inclusion of viscosity and heat conductivity, a few works have been performed based on the gas-kinetic scheme [6,7,8]. However, to the best of the author’s knowledge, the study of the cross-scale modeling and computation of multicomponent gas dynamics under an external force field is very limited.

In recent years, the unified gas-kinetic scheme (UGKS) has been developed for the simulation of multiscale gaseous flow [9,10]. Based on the Boltzmann model equation, the UGKS uses an analytical integral solution in the construction of numerical flux functions. The coupled modeling of particle transport and the collision of the evolution solution guarantees the multiscale nature of the method. For the gas dynamic system related to an external force, in order to develop a well-balanced gas-kinetic scheme, it is important to take the external force effect into the flux transport across a cell interface accurately. Based on this idea, a well-balanced unified gas-kinetic scheme for single-component flow [11] has been proposed. In this paper, a similar methodology is used in the flux function for the further development of the unified gas-kinetic scheme for a gas mixture. It is worth mentioning that, due to the versatility of kinetic theory, it is natural to develop kinetic schemes for other multi-particle systems, including shallow water equations [12], radiative transfer [13], weakly coupled plasma physics [14], etc.

This paper is organized as follows. Section 2 is a brief introduction of the kinetic theory of multicomponent gases and the asymptotic analysis of the current Boltzmann model. Section 3 presents the construction of the well-balanced unified gas-kinetic scheme for a gas mixture under an external force field. Section 4 includes numerical examples to demonstrate the performance of the scheme. The last section is the conclusion.

## 2. Kinetic Theory

### 2.1. Boltzmann Equation and Relaxation Model

The kinetic theory describes the evolution of gases in a statistical fashion. The Boltzmann equation for single-component flows is written as

$$\frac{\partial f}{\partial t}+u_{i}\frac{\partial f}{\partial x_{i}}+\phi_{i}\frac{\partial f}{\partial u_{i}}=Q\left(f,f\right),$$

where $u_{i}=\left(u,v,w\right)$ is the particle velocity, $\phi_{i}$ is the external forcing term, and Q(f,f) denotes the two-body collision term. Here, Einstein’s summation convention is adopted for tensor operations. The above equation can be extended to a gas mixture, where the evolution equation for the distribution function of each species *s* is written as

(1) $$\frac{\partial f_{s}}{\partial t}+u_{i}\frac{\partial f_{s}}{\partial x_{i}}+\phi_{i}\frac{\partial f_{s}}{\partial u_{i}}=Q_{s}\left(f,f\right).$$

The collision term can be written as

(2) $$Q_{s}\left(f,f\right)=\sum_{r=1}^{N}Q_{sr}\left(f_{s},f_{r}\right)=\sum_{r=1}^{N}\int_{R^{3}}\int_{S^{2}}\left(f_{s}^{\prime }f_{r}^{\prime }-f_{s}f_{r}\right)g_{sr}\sigma_{sr}d\Omega du_{ri},$$

where $f^{\prime }$ is the post-collision distribution and *r* is the index of different gas species. The term $g_{sr}$ is the relative speed of two molecular classes, and $\sigma_{sr}d\Omega$ is the differential cross-section for the collision specified. Here, $Q_{ss}\left(f_{s},f_{s}\right)$ is called the self-collision term and $Q_{sr}\left(f_{s},f_{r}\right)$ is the cross-collision term.

Due to the complexity of the collision integral in Equation (2), simplified kinetic models have been proposed for single-component gas evolution [15]. Such a model is expected to satisfy some key structures of the original Boltzmann equation, such as positivity, correct exchange coefficients, entropy inequality, and indifferentiability. Here, we introduce a BGK-type model proposed by Andries, Aoki, and Perthame (AAP) [16], which could satisfy all the properties required above. In the AAP model, a single collision operator for species *s* is defined as

(3) $$Q_{s}\left(f\right)=\frac{f_{s}^{+}-f_{s}}{\tau_{s}}.$$

Here, the equilibrium state is defined based on modified macroscopic variables, i.e.,

(4) $$f_{s}^{+}=n_{s}{\left(\frac{m_{s}}{2\pi k_{B}T_{s}^{\prime }}\right)}^{3/2}\exp\left(-\frac{m_{s}}{2k_{B}T_{s}^{\prime }}{\left(u_{s}-U_{s}^{\prime }\right)}^{2}\right),$$

where $\left\{U_{s}^{\prime },T_{s}^{\prime }\right\}$ is the modified bulk velocity and temperature, $n_{s}$ is the number density, $m_{s}$ is the molecular mass, and $k_{B}$ is the Boltzmann constant. The determination of modified temperature $T_{s}^{\prime }$ and velocity $U_{s}^{\prime }$ can be found in [17] to take into account the interaction among different gas species:

(5) $$\begin{array}{cc} \; & U_{si}^{\prime }=U_{si}+\tau_{s}\sum_{r\neq s}2\frac{\rho_{r}}{m_{s}+m_{r}}\theta_{sr}\left(U_{ri}-U_{si}\right),\\ \; & \frac{3}{2}k_{B}T_{s}^{\prime }=\frac{3}{2}k_{B}T_{s}-\frac{m_{s}}{2}{\left(U_{s}^{\prime }-U_{s}\right)}^{2}\\ \; & +\tau_{s}\sum_{r\neq s}4m_{s}\frac{\rho_{r}}{{\left(m_{s}+m_{r}\right)}^{2}}\theta_{sr}\left(\frac{3}{2}k_{B}T_{r}-\frac{3}{2}k_{B}T_{s}+\frac{m_{r}}{2}{\left(U_{r}-U_{s}\right)}^{2}\right), \end{array}$$

where ρ=mn is the mass density.

The collision frequency is determined by

(6) $$\frac{1}{\tau_{s}}=\beta \sum_{r}\theta_{sr}n_{r},$$

where β can be chosen as either theunit for simplicity or to coincide with the collision time of the single-component gas when all components are the same species. The parameter $\theta_{sr}$ is defined as

(7) $$\theta_{sr}=\frac{4\sqrt{\pi }}{3}{\left(\frac{2k_{B}T_{s}}{m_{s}}+\frac{2k_{B}T_{r}}{m_{r}}\right)}^{1/2}{\left(\frac{d_{s}+d_{r}}{2}\right)}^{2},$$

for the hard sphere model and

(8) $$\theta_{sr}=0.422\pi \left(\frac{a_{sr}\left(m_{s}+m_{r}\right)}{m_{s}m_{r}}\right),$$

for the Maxwell molecule, where $d_{s},d_{r}$ are the molecular diameters and $a_{sr}$ is the proportionality of the intermolecular force.

With the defined collision operator, the BGK-type kinetic model equation can be written as

(9) $$\frac{\partial f_{s}}{\partial t}+u_{i}\frac{\partial f_{s}}{\partial x_{i}}+\phi_{i}\frac{\partial f_{s}}{\partial u_{i}}=\frac{f_{s}^{+}-f_{s}}{\tau_{s}}.$$

### 2.2. Asymptotic Analysis

The macroscopic conservative flow variables can be obtained from the moments of the particle distribution function, i.e.,

$$W_{s}=\left(\begin{array}{c} \rho_{s}\\ \rho_{s}U_{si}\\ \rho_{s}E_{s} \end{array}\right)=\int_{R^{3}}f_{s}\psi d\Xi ,$$

where $\psi ={\left(m_{s},m_{s}u_{i},\frac{1}{2}m_{s}u_{i}u_{i}\right)}^{T}$ is a vector of moments for collision invariants and dΞ=dudvdw. Taking the moments of Equation (9) yields the balance laws of density, momentum, and energy in each species *s*, i.e.,

(10) $$\begin{array}{cc} \; & \frac{\partial \rho_{s}}{\partial t}+\frac{\partial \rho_{s}U_{si}}{\partial x_{i}}=0,\\ \; & \frac{\partial \rho_{s}U_{si}}{\partial t}+\frac{\partial \rho_{s}U_{si}U_{sj}}{\partial x_{j}}+\frac{\partial T_{sij}}{\partial x_{j}}=\rho_{s}\phi_{i}+\int_{R^{3}}u_{i}Q_{s}\left(f\right)d\Xi ,\\ \; & \frac{\partial \rho_{s}E_{s}}{\partial t}+\frac{\partial \rho_{s}E_{s}U_{si}}{\partial x_{i}}+\frac{\partial (T_{sij}U_{sj}+q_{si})}{\partial x_{i}}=\rho_{s}U_{si}\phi_{i}+\int_{R^{3}}\frac{1}{2}u_{i}u_{i}Q_{s}\left(f\right)d\Xi . \end{array}$$

The term $T_{ij}$ is the stress tensor, and $q_{i}$ is the heat flux. It is noticeable that, due to the momentum and energy exchanges among different species in the mixture, the collision integrals $\int u_{i}Q_{s}\left(f,f\right)d\Xi$ and $\int \frac{1}{2}u_{i}u_{i}Q_{s}\left(f,f\right)d\Xi$ are no longer equal to zero, while the total density, momentum, and energy are still conserved in the flow evolution. Therefore, summing up the above equations, we can obtain

(11) $$\begin{array}{cc} \; & \frac{\partial \rho_{s}}{\partial t}+\frac{\partial \rho_{s}U_{i}}{\partial x_{i}}=-\frac{\partial J_{si}}{\partial x_{i}},\\ \; & \frac{\partial \rho U_{i}}{\partial t}+\frac{\partial \rho U_{i}U_{j}}{\partial x_{j}}+\frac{\partial T_{ij}}{\partial x_{j}}=\rho \phi_{i},\\ \; & \frac{\partial \rho E}{\partial t}+\frac{\partial \rho EU_{i}}{\partial x_{i}}+\frac{\partial (T_{ij}U_{j}+q_{i})}{\partial x_{i}}=\rho U_{i}\phi_{i}. \end{array}$$

where $J_{si}=\int \left(u_{i}-U_{i}\right)f_{s}d\Xi$. As shown in [16], by inserting the Chapman–Enskog expansion, e.g., the zeroth-order approximation:

$$f_{s}≃f_{s}^{+}+O\left(\tau_{s}\right),$$

and the first-order approximation:

$$f_{s}≃f_{s}^{+}-\tau_{s}\left(\partial_{t}f_{s}^{+}+u_{i}\partial_{x_{i}}f_{s}^{+}\right)+O\left(\tau_{s}^{2}\right),$$

into the determination of the stress tensor and heat flux, one can derive the Euler and Navier–Stokes equations, respectively.

For multicomponent flows, the mass transfer is another important topic. Here, we used diffusive scaling to illustrate the mechanism of mass transfer and diffusion in the current model. We introduce dimensionless variables denoted with asterisks:

$$t=t^{*}t_{0},\;x=x^{*}x_{0},\;u_{i}=u_{i}^{*}u_{0},\;f=f^{*}f_{0},$$

where $t_{0}$ is the reference time scale, $x_{0}$ is the reference length scale, and so on. With the dimensionless terms plugged into Equation (9), we obtain (after immediately dropping the asterisks)

$$\mathrm{St}\frac{\partial f_{s}}{\partial t}+u_{i}\frac{\partial f_{s}}{\partial x_{i}}+\phi_{i}\frac{\partial f_{s}}{\partial u_{i}}=\frac{1}{\mathrm{Kn}}Q_{s}\left(f\right),$$

where $\mathrm{St}=x_{0}/u_{0}t_{0}$ is the Strouhal number and Kn is the Knudsen number. In the diffusive limit, we assume St≃Kn=ϵ. The stiff term 1/ϵ on the right-hand side implies that the limiting solution $\lim_{\varepsilon \to 0}f_{s}^{\varepsilon }$ is close to the local equilibrium. We make this assumption and compute the moment system in the same way as Equations (10) and (11), which yields

$$\begin{array}{cc} \; & \epsilon \frac{\partial n_{s}^{\epsilon }}{\partial t}+\epsilon \frac{\partial (n_{s}^{\epsilon }U_{si}^{\epsilon })}{\partial x_{i}}=0,\\ \; & \epsilon^{2}\frac{\partial \rho_{s}^{\epsilon }U_{si}^{\epsilon }}{\partial t}+\epsilon^{2}\frac{\partial (\rho_{s}^{\epsilon }U_{si}^{\epsilon }U_{sj}^{\epsilon })}{\partial x_{i}}+\frac{\partial (n_{s}^{\epsilon }k_{B}T^{\epsilon })}{\partial x_{i}}=\frac{1}{\epsilon }\int m_{s}u_{i}Q_{s}\left(f^{\epsilon }\right)d\Xi +\epsilon^{2}\rho_{s}^{\epsilon }\phi_{i}. \end{array}$$

For simplicity, here, we adopt the number density in the continuity equation. Truncating the above equations at the leading order in ϵ leads to

$$\begin{array}{cc} \; & \frac{\partial n^{\epsilon }}{\partial t}+\frac{\partial n^{\epsilon }U_{i}^{\epsilon }}{\partial x_{i}}=0,\\ \; & \frac{\partial n_{s}^{\epsilon }k_{B}T^{\epsilon }}{\partial x_{i}}=\frac{1}{\epsilon }\int m_{s}u_{i}Q_{s}\left(f^{\epsilon }\right)d\Xi . \end{array}$$

If the isothermal assumption is made, the second equation with ϵ→0 reduces to

(12) $$\begin{array}{cc} \frac{\partial n_{s}^{\epsilon }}{\partial x_{i}} & =\frac{1}{\epsilon k_{B}T^{\epsilon }}\int m_{s}u_{i}Q_{s}\left(f^{\epsilon }\right)d\Xi =\frac{U_{si}^{\prime }-U_{si}}{\epsilon k_{B}T}\\ \; & =\sum_{r\neq s}\frac{\left(U_{ri}-U_{si}\right)}{D_{ij}}, \end{array}$$

where the coefficients $D_{ij}$ are determined by the collision time in Equation (6) and the interaction model in Equation (5). Equation (12) is exactly the Maxwell–Stefan diffusion law [18]. As analyzed, even though the Maxwell–Stefan theory is basically understood as a more generalized law than Fick’s law to describe mass transfer, its applicability is mainly limited to the continuum limit and thermodynamic equilibrium. To study the mass and heat transfer in multiscale and non-equilibrium fluids, we must resort to reliable numerical methods, which is the core task in the next section.

## 3. Numerical Algorithm

### 3.1. Construction of Interface Distribution Function

The key ingredient in the UGKS is the integral solution constructed from the BGK-type relaxation model. Here, we used the one-dimensional case to illustrate the construction of the numerical algorithm first. Without loss of generality, we assumed the interface between two neighbor cells $x_{i+1/2}=0$ and $t^{n}=0$. Given a local constant collision time $\tau_{s}$, the integral solution of Equation (9) along the characteristic line is written as

(13) $$\begin{array}{cc} f_{s}\left(0,t,u_{k}\right)= & \frac{1}{\tau_{s}}\int_{0}^{t}f_{s}^{+}\left(x^{\prime },t^{\prime },u_{k}^{\prime }\right)e^{-(t-t^{\prime })/\tau }dt^{\prime }\\ \; & +e^{-t/\tau }{\left(f_{s}\right)}_{0}\left(x^{0},0,u_{k}^{0}\right), \end{array}$$

where $x_{i}^{\prime }=x_{i}-u_{i}^{\prime }\left(t-t^{\prime }\right)-\frac{1}{2}\phi_{i}{\left(t-t^{\prime }\right)}^{2}$ denotes the particle trajectories in physical space, $u_{i}^{\prime }=u_{i}-\phi_{i}\left(t-t^{\prime }\right)$ is the particle velocities under acceleration, $\left(x^{0},u^{0}\right)$ is the initial location in the phase space for the particle that passes through the cell interface at time *t*, and ${\left(f_{s}\right)}_{0}$ is the particle distribution function of species *s* at the beginning of the *n*-th time step.

In the numerical algorithm, the initial gas distribution function ${\left(f_{s}\right)}_{0}$ of each gas component *s* around the cell interface $x_{i+1/2}$ is reconstructed as follows:

(14) $${\left(f_{s}\right)}_{0}\left(x,0,u_{k}\right)=\left\{\begin{array}{c} {\left(f_{s}\right)}_{i+1/2,k}^{L}+{\left(\sigma_{s}\right)}_{i,k}x,\;x\leq 0,\\ {\left(f_{s}\right)}_{i+1/2,k}^{R}+{\left(\sigma_{s}\right)}_{i+1,k}x,\;x>0, \end{array}\right.$$

where ${\left(f_{s}\right)}_{i+1/2,k}^{L,R}$ are the reconstructed values of the initial distribution functions from both sides of the cell interface. Based on the reconstructed distribution functions, the macroscopic conservative variables at a cell interface can be evaluated through

$$W_{s}=\sum_{u_{k}>0}f_{i+1/2,k}^{L}\psi d\Xi +\sum_{u_{k}<0}f_{i+1/2,k}^{R}\psi d\Xi ,$$

which can be used to determine the modified macroscopic variables $W_{s}^{\prime }$ in Equation (5) and the equilibrium distribution ${\left(f_{s}\right)}_{0}^{+}$ in Equation (4).

For the second part of the integral solution, the equilibrium distribution is expanded in space and time around a cell interface as

(15) $$f_{s}^{+}={\left(f_{s}\right)}_{0}^{+}\left[1+\left(1-H\left[x\right]\right)a^{L}x+H\left[x\right]a^{R}x+At\right],$$

where H[x] is the Heaviside step function. Here, $a_{s}^{L},a_{s}^{R}$, and $A_{s}$ are from the Taylor expansion of a Maxwellian:

$$\begin{array}{cc} \; & a_{s}^{L,R}=a_{1}^{L,R}+a_{2}^{L,R}u+a_{3}^{L,R}\frac{1}{2}u^{2}=a_{\alpha }^{L,R}\psi_{\alpha },\\ \; & A_{s}=A_{1}+A_{2}u+A_{3}\frac{1}{2}u^{2}=A_{\alpha }\psi_{\alpha }. \end{array}$$

The spatial slopes $a_{s}^{L},a_{s}^{R}$ can be obtained from the slopes of modified conservative variables on both sides of a cell interface:

$${\left(\frac{\partial W_{s}^{\prime }}{\partial x}\right)}^{L}=\int a_{s}^{L}{\left(f_{s}\right)}_{0}^{+}\psi d\Xi ,\;{\left(\frac{\partial W_{s}^{\prime }}{\partial x}\right)}^{R}=\int a_{s}^{R}{\left(f_{s}\right)}_{0}^{+}\psi d\Xi .$$

The time derivative $A_{s}$ of $f_{s}^{+}$ is related to the temporal variation of conservative flow variables:

$$\frac{\partial W_{s}^{\prime }}{\partial t}=\int A_{s}{\left(f_{s}\right)}_{0}^{+}\psi d\Xi ,$$

and it can be calculated via the time derivative of the overall compatibility condition for the gas mixture:

$$\frac{d}{dt}\int \sum_{r=1}^{s}\left(f_{r}^{+}-f_{r}\right)\psi d\Xi {|}_{x=0,t=0}=0.$$

Once we determine all the coefficients, the integral solution can be rewritten as

(16) $$\begin{array}{cc} f_{s}\left(0,t,u_{k}\right)= & \left(1-e^{-t/\tau }\right){\left(f_{s}\right)}_{0}^{+}\\ \; & +\left(\tau \left(-1+e^{-t/\tau }\right)+te^{-t/\tau }\right)a_{s}^{L,R}u_{k}{\left(f_{s}\right)}_{0}^{+}\\ \; & -\left[\tau \left(\tau \left(-1+e^{-t/\tau }\right)+te^{-t/\tau }\right)+\frac{1}{2}t^{2}e^{-t/\tau }\right]a_{s}^{L,R}\phi_{x}{\left(f_{s}\right)}_{0}^{+}\\ \; & +\tau \left(t/\tau -1+e^{-t/\tau }\right)A_{s}{\left(f_{s}\right)}_{0}^{+}\\ \; & +e^{-t/\tau }\left[\left({\left(f_{s}\right)}_{i+1/2,k^{0}}^{L}+\left(-\left(u_{k}-\phi_{x}t\right)t-\frac{1}{2}\phi_{x}t^{2}\right){\left(\sigma_{s}\right)}_{i,k^{0}}\right)H\left[u_{k}-\frac{1}{2}\phi_{x}t\right]\right.\\ \; & \left.+\left({\left(f_{s}\right)}_{i+1/2,k^{0}}^{R}+\left(-\left(u_{k}-\phi_{x}t\right)t-\frac{1}{2}\phi_{x}t^{2}\right){\left(\sigma_{s}\right)}_{i+1,k^{0}}\right)\left(1-H\left[u_{k}-\frac{1}{2}\phi_{x}t\right]\right)\right], \end{array}$$

from which we can evaluate the numerical fluxes for both the particle distribution function and macroscopic conservative variables.

### 3.2. Two-Dimensional Case

Following the integral solution of the relaxation model, it is natural to extended the UGKS to the multidimensional case. Under the force $\phi =(\phi_{x},\phi_{y})$, the integral solution of the AAP kinetic model in the two-dimensional Cartesian coordinate system is written as

(17) $$\begin{array}{cc} f_{s}\left(x,y,t,u,v\right)= & \frac{1}{\tau }\int_{t^{n}}^{t}f_{s}^{+}\left(x^{\prime },y^{\prime },t^{\prime },u^{\prime },v^{\prime }\right)e^{-(t-t^{\prime })/\tau }dt^{\prime }\\ \; & +e^{-(t-t^{n})/\tau }{\left(f_{s}\right)}_{0}^{n}\left(x^{n},y^{n},t^{n},u^{n},v^{n}\right), \end{array}$$

where $x^{\prime }=x-u^{\prime }\left(t-t^{\prime }\right)-\frac{1}{2}\phi_{x}{\left(t-t^{\prime }\right)}^{2},\;y^{\prime }=y-v^{\prime }\left(t-t^{\prime }\right)-\frac{1}{2}\phi_{y}{\left(t-t^{\prime }\right)}^{2},\;u^{\prime }=u-\phi_{x}\left(t-t^{\prime }\right)$, and $v^{\prime }=v-\phi_{y}\left(t-t^{\prime }\right)$. For simplicity, we will drop the subscript *s* to denote a single gas species.

In the unified scheme, at the center of a cell interface $\left(x_{i+1/2},y_{j}\right)$, the solution $f_{i+1/2,j,k,l}$ is constructed from the integral solution Equation (17). With the notations $x_{i+1/2}=0,\;y_{j}=0$ at $t^{n}=0$, the time-dependent interface distribution function for species *s* goes to

$$\begin{array}{cc} f(0,0,t,u_{k},v_{l})= & \frac{1}{\tau }\int_{0}^{t}f^{+}\left(x^{\prime },y^{\prime },t^{\prime },u_{k}^{\prime },v_{l}^{\prime }\right)e^{-(t-t^{\prime })/\tau }dt^{\prime }\\ \; & +e^{-t/\tau }f_{0}\left(-u_{k}t+\frac{1}{2}\phi_{x}t^{2},-v_{l}t+\frac{1}{2}\phi_{y}t^{2},0,u_{k}-\phi_{x}t,v_{l}-\phi_{y}t\right). \end{array}$$

To second-order accuracy, the initial gas distribution function $f_{0}$ is reconstructed as

(18) $$f_{0}\left(x,y,0,u_{k},v_{l}\right)=\left\{\begin{array}{c} f_{i+1/2,j,k,l}^{L}+\sigma_{i,j,k,l}x+\theta_{i,j,k,l}y,\;x\leq 0,\\ f_{i+1/2,j,k,l}^{R}+\sigma_{i+1,j,k,l}x+\theta_{i+1,j,k,l}y,\;x>0, \end{array}\right.$$

where $f_{i+1/2,j,k,l}^{L}$ and $f_{i+1/2,j,k,l}^{R}$ are the reconstructed initial distribution functions on the left- and right-hand sides of a cell interface. The slope of *f* at the (i,j)-thcell and the (k,l)-thdiscretized velocity point in the *x*-direction and *y*-direction are denoted by $\sigma_{i,j,k,l}$ and $\theta_{i,j,k,l}$.

The modified equilibrium distribution function around a cell interface is constructed as

$$f^{+}=f_{0}^{+}\left[1+\left(1-H\left[x\right]\right)a^{L}x+H\left[x\right]a^{R}x+by+At\right],$$

where $f_{0}^{+}$ is the equilibrium distribution at (x=0,t=0). The coefficients above can be determined in the same way as the one-dimensional case.

The time-dependent interface distribution function is written as

(19) $$\begin{array}{cc} f(0,0,t,u_{k},v_{l})= & \left(1-e^{-t/\tau }\right)f_{0}^{+}\\ \; & +\left(\tau \left(-1+e^{-t/\tau }\right)+te^{-t/\tau }\right)a^{L,R}u_{k}f_{0}^{+}\\ \; & -\left[\tau \left(\tau \left(-1+e^{-t/\tau }\right)+te^{-t/\tau }\right)+\frac{1}{2}t^{2}e^{-t/\tau }\right]a^{L,R}\phi_{x}f_{0}^{+}\\ \; & +\left(\tau \left(-1+e^{-t/\tau }\right)+te^{-t/\tau }\right)bv_{l}f_{0}^{+}\\ \; & -\left[\tau \left(\tau \left(-1+e^{-t/\tau }\right)+te^{-t/\tau }\right)+\frac{1}{2}t^{2}e^{-t/\tau }\right]b\phi_{y}f_{0}^{+}\\ \; & +\tau \left(t/\tau -1+e^{-t/\tau }\right)Af_{0}^{+}\\ \; & +e^{-t/\tau }\left[\left(f_{i+1/2,k^{0},l^{0}}^{L}+\left(-\left(u_{k}-\phi_{x}t\right)t-\frac{1}{2}\phi_{x}t^{2}\right)\sigma_{i,k^{0},l^{0}}\right.\right.\\ \; & \left.\left.+\left(-\left(v_{l}-\phi_{y}t\right)t-\frac{1}{2}\phi_{y}t^{2}\right)\theta_{i,k^{0},l^{0}}\right)H\left[u_{k}-\frac{1}{2}\phi_{x}t\right]\right.\\ \; & \left.+\left(f_{i+1/2,k^{0},l^{0}}^{R}+\left(-\left(u_{k}-\phi_{x}t\right)t-\frac{1}{2}\phi_{x}t^{2}\right)\sigma_{i+1,k^{0},l^{0}}\right.\right.\\ \; & \left.\left.+\left(-\left(v_{l}-\phi_{y}t\right)t-\frac{1}{2}\phi_{y}t^{2}\right)\theta_{i+1,k^{0},l^{0}}\right)\left(1-H\left[u_{k}-\frac{1}{2}\phi_{x}t\right]\right)\right]. \end{array}$$

The extension of the above method to the three-dimensional case is straightforward.

### 3.3. Update Algorithm

With the cell-averaged distribution function for species *s* in the gas mixture:

$$f_{x_{i},y_{j},t^{n},u_{k},v_{l}}=f_{i,j,k,l}^{n}=\frac{1}{\Omega_{i,j}\left(x,y\right)\Omega_{k,l}\left(u,v\right)}\int_{\Omega_{i,j}}\int_{\Omega_{k,l}}f\left(x,y,t^{n},u,v\right)dxdydudv,$$

the direct modeling for its evolution gives the conservation laws of macroscopic variables and the particle distribution function in a discretized space:

(20) $$\begin{array}{cc} W_{i,j}^{n+1}=\; & W_{i,j}^{n}+\frac{1}{\Omega_{i,j}}\int_{t^{n}}^{t^{n+1}}\sum_{i=1}\Delta L_{i}\cdot F_{i}dt\\ \; & +\frac{1}{\Omega_{i,j}}\int_{t^{n}}^{t^{n+1}}\int_{\Omega_{i,j}}Q_{i,j}dxdydt+\frac{1}{\Omega_{i,j}}\int_{t^{n}}^{t^{n+1}}\int_{\Omega_{i,j}}G_{i,j}dxdydt, \end{array}$$

(21) $$\begin{array}{cc} f_{i,j,k,l}^{n+1}= & f_{i,j,k,l}^{n}+\frac{1}{\Omega_{i,j}}\int_{t^{n}}^{t^{n+1}}\sum_{i=1}u_{i}{\hat{f}}_{i}\left(t\right)\Delta L_{i}dt\\ \; & +\frac{1}{\Omega_{i,j}}\int_{t^{n}}^{t^{n+1}}\int_{\Omega_{i,j}}Q\left(f\right)dxdydt+\frac{1}{\Omega_{i,j}}\int_{t^{n}}^{t^{n+1}}\int_{\Omega_{i,j}}G\left(f\right)dxdydt, \end{array}$$

where $F_{i}$ is the flux of conservative variables across the cell interface $\Delta L_{i}=\Delta L_{i}n_{i}$, ${\hat{f}}_{i}$ is the time-dependent gas distribution function at the cell interface, and $\Delta L_{i}$ is the cell interface length. $Q_{i,j}$, Q(f) are the source terms from intermolecular collisions, and $G_{i,j}$, G(f) are the external forcing terms:

(22) $$\begin{array}{cc} \; & Q\left(f\right)=\frac{f_{i,j,k,l}^{+}-f_{i,j,k,l}^{n+1/2}}{\tau },\\ \; & Q_{i,j}=\int_{\Omega_{k,l}}\frac{f_{i,j,k,l}^{+}-f_{i,j,k,l}^{n+1/2}}{\tau }\psi dudv, \end{array}$$

(23) $$\begin{array}{cc} \; & G\left(f\right)=-\phi_{x}\frac{\partial }{\partial u}f_{i,j,k,l}^{n+1/2}-\phi_{y}\frac{\partial }{\partial v}f_{i,j,k,l}^{n+1/2},\\ \; & G_{i,j}=\int_{\Omega_{k,l}}\left(-\phi_{x}\frac{\partial }{\partial u}f_{i,j,k,l}^{n+1/2}-\phi_{y}\frac{\partial }{\partial v}f_{i,j,k,l}^{n+1/2}\right)\psi dudv. \end{array}$$

In the UGKS, we use the semi-implicit method to model the collision term and the fully implicit one for the external forcing term:

(24) $$\begin{array}{cc} f_{i,j,k,l}^{n+1}= & f_{i,j,k,l}^{n}+\frac{1}{\Omega_{i,j}}\left(F_{i-1/2,j,k,l}-F_{i+1/2,j,k,l}\right)+\frac{1}{\Omega_{i,j}}\left(F_{i,j-1/2,k,l}-F_{i,j+1/2,k,l}\right)\\ \; & +\frac{\Delta t}{2}\left(\frac{f_{i,j,k,l}^{+(n+1)}-f_{i,j,k,l}^{n+1}}{\tau^{n+1}}+\frac{f_{i,j,k,l}^{+(n)}-f_{i,j,k,l}^{n}}{\tau^{n}}\right)-\Delta t\left(\phi_{x}\frac{\partial }{\partial u}f_{i,j,k,l}^{n+1}+\phi_{y}\frac{\partial }{\partial v}f_{i,j,k,l}^{n+1}\right). \end{array}$$

In order to update the gas distribution function implicitly, we solve Equation (20) first, and its solution can be used for the construction of the equilibrium state in Equation (24) at $t^{n+1}$. In the current scheme, the collision term for macroscopic variables is treated as

(25) $$\frac{1}{\Omega_{i,j}}\int_{t^{n}}^{t^{n+1}}\int_{\Omega_{i,j}}Q_{i,j}dxdydt=\frac{\Delta t}{\tau }\left[{\left(W^{\prime }\right)}^{n}-W^{n}\right],$$

where ${\left(W^{\prime }\right)}^{n}$ is the modified macroscopic conservative variable. For the external forcing source, we adopted the numerical methodology proposed by Slyz and Prendergast [19], where the energy source term from the external force can be absorbed into the energy flux as $\Phi F^{\rho }$, where $F^{\rho }$ is the mass flux, to ensure the accurate conservation of energy. A similar implicit upwind update as [11] was adopted to update the particle distribution function.

With the help of the implicit update algorithm, the time step is not restricted by the collision time and is fully determined by the CFL condition:

(26) $$\Delta t=\mathrm{CFL}\min\left\{\frac{\Delta x\Delta y}{u_{max}\Delta y+v_{max}\Delta x},\frac{\Delta u\Delta v}{|\phi_{x}\left|\Delta v+\right|\phi_{y}|\Delta u}\right\},$$

where CFL is the CFL number, $\{u_{max}=\max(|u_{k}|),v_{max}=\max(|v_{l}\left|)\right\}$ is the largest discretized particle velocity of all gas components in the *x*- and *y*-directions, and {Δu,Δv} is the distance between two neighboring velocity points.

### 3.4. Analysis on the Well-Balanced Property

In this part, we prove the well-balanced property of the current scheme theoretically. In the continuum regime with intensive intermolecular collisions, the fluid element picture can be used to describe the bulk property of flow transport. We adopted the one-dimensional Euler equations for multicomponent flow under force field Φ, i.e.,

$$\begin{array}{cc} \; & {\left(\rho_{1}\right)}_{t}+{\left(\rho_{1}U\right)}_{x}=0,\\ \; & {\left(\rho_{2}\right)}_{t}+{\left(\rho_{2}U\right)}_{x}=0,\\ \; & {\left(\rho U\right)}_{t}+{\left(\rho U^{2}+p\right)}_{x}=\rho \phi_{x},\\ \; & {\left(\rho E\right)}_{t}+{\left(\left(\rho E+p\right)U\right)}_{x}=\rho U\phi_{x}. \end{array}$$

where ρ,ρU,ρE,p are the total density, momentum, energy, and pressure. It is clear that the equations above allow a simply hydrostatic solution where the macroscopic flow is absent and the pressure gradient is balanced by the density stratification:

$$\rho =\rho \left(x\right)=\rho_{1}\left(x\right)+\rho_{2}\left(x\right),U=0,p_{x}={\left(p_{1}\right)}_{x}+{\left(p_{2}\right)}_{x}=\left(\rho_{1}+\rho_{2}\right)\phi_{x}.$$

Given a constant force field $\phi_{x}$, the above solution can be rewritten as

(27) $$\rho =\rho_{1}+\rho_{2}=\rho_{0}\exp\left(\frac{\phi_{x}x}{RT}\right),U=0,p=p_{1}+p_{2}=p_{0}\exp\left(\frac{\phi_{x}x}{RT}\right),$$

where *R* is the gas constant. Such a steady-state solution needs to be maintained due to the exact balance between the gravitational source term and the inhomogeneous flux function for each gas component in the mixture, i.e.,

(28) $$\frac{1}{\Delta x}\int_{t^{n}}^{t^{n+1}}\left(F_{i-1/2}-F_{i+1/2}\right)dt+\frac{1}{\Delta x}\int_{t^{n}}^{t^{n+1}}\int_{x_{1-1/2}}^{x_{i+1/2}}G_{i}dt=0.$$

In the hydrodynamic scale where Δt≫τ, under hydrostatic balance, the intensive particle collision will converge the interface distribution function in Equation (16) to

(29) $$f_{i+1/2}=f_{0}^{+}-\tau auf_{0}^{+}-\tau^{2}a\phi_{x}f_{0}^{+}.$$

The velocity moments $\int u^{\alpha }f_{0}^{+}du=\rho \langle u^{\alpha }\rangle$ of the above solution can be evaluated analytically. The coefficient *a* in Equation (29) can be determined by the slopes of conservative variables:

$$\begin{array}{cc} \; & a_{3}=\frac{4{\left(\lambda_{0}^{\prime }\right)}^{2}}{\left(K+1\right)\rho_{0}}\left[2{\left(\rho E^{\prime }\right)}_{x}+\left({\left(U_{0}^{\prime }\right)}^{2}-\frac{K+1}{2\lambda_{0}^{\prime }}\right)\rho_{x}-2{\bar{U}}_{0}{\left(\rho U^{\prime }\right)}_{x}\right],\\ \; & a_{2}=\frac{2\lambda_{0}^{\prime }}{\rho_{0}}\left[{\left(\rho U^{\prime }\right)}_{x}-U_{0}^{\prime }\rho_{x}\right]-U_{0}^{\prime }a_{3},\\ \; & a_{1}=\frac{1}{\rho_{0}}\rho_{x}-U_{0}^{\prime }a_{2}-\frac{1}{2}\left({\left(U_{0}^{\prime }\right)}^{2}+\frac{K+1}{2\lambda_{0}^{\prime }}\right)a_{3}, \end{array}$$

where $\left(U_{0}^{\prime },\lambda_{0}^{\prime }\right)$ are the modified primitive variables in Equation (5). In the isothermal and static case, the above equation can be further reduced to

$$a_{1}=\frac{1}{\rho_{0}}\frac{\partial \rho }{\partial x},a_{2}=a_{3}=0.$$

Therefore, the fluxes of density, momentum, and energy can be obtained via $F_{i+1/2}=\int uf_{i+1/2}\psi du$, i.e.,

$$\begin{array}{cc} \; & F_{i+1/2}^{\rho }=0,\\ \; & F_{i+1/2}^{\rho U}=\frac{\rho_{i+1/2}}{2\lambda^{\prime }},\\ \; & F_{i+1/2}^{\rho E}=0. \end{array}$$

At the same time, the source term in Equation (28) is

$$G_{i}=\int -\phi_{x}f_{u}\psi du.$$

The source term from the external force can be integrated as

$$\begin{array}{cc} \; & G^{\rho }=0,\\ \; & G^{\rho U}=\rho \phi_{x},\\ \; & G^{\rho E}=\rho U\phi_{x}=0. \end{array}$$

For the momentum balance equation, we can use the exponential density distribution in Equation (27) to check the well-balanced relationship in Equation (28). As the temperature is uniform in the flow domain, the modified $\lambda^{\prime }$ is equivalent to each component’s λ, and the balance relationship is

$$\int_{x_{i-1/2}}^{x_{i+1/2}}G_{i}^{\rho U}dx=\int_{x_{i-1/2}}^{x_{i+1/2}}\rho \phi_{x}dx=RT\left(\rho_{i+1/2}-\rho_{i-1/2}\right)=-\left(F_{i-1/2}-F_{i+1/2}\right),$$

from which we can see that the well-balanced property is precisely satisfied in the current scheme.

In another limit of the Knudsen regime, where τ≫Δt, the current method recovers a purely upwind method:

$$f_{i+1/2,k}=\left\{\begin{array}{c} f_{i+1/2,k^{0}}^{L}+\left(-u_{k}t+\frac{1}{2}\phi_{x}t^{2}\right)\sigma_{i,k^{0}},\;u_{k}-\frac{1}{2}\phi_{x}t\geq 0,\\ f_{i+1/2,k^{0}}^{R}+\left(-u_{k}t+\frac{1}{2}\phi_{x}t^{2}\right)\sigma_{i+1,k^{0}},\;u_{k}-\frac{1}{2}\phi_{x}t<0. \end{array}\right.$$

With the forcing effect on each particle, the particle distribution function will become distorted in the velocity space, and the deviation from the equilibrium state is restricted with the particle collision time τ. There is no more isothermal equilibrium due to the non-equilibrium heat transfer induced by the force field, as analyzed in [20]. In this case, the good hydrostatic balance is only a coarse-grained concept based on statistical averaging.

### 3.5. Summary of the Algorithm

**Figure 1:**
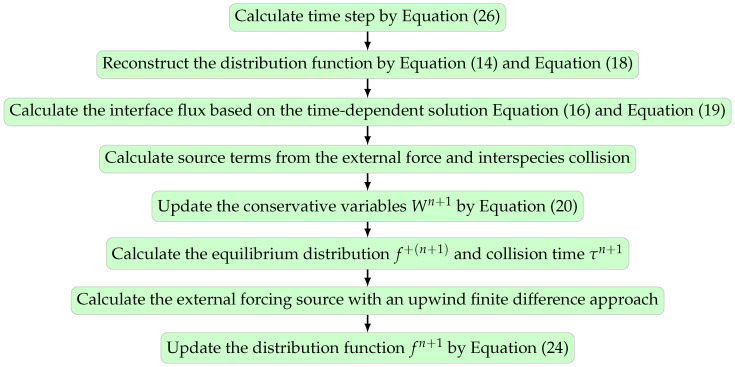
Numerical algorithm of the UGKS.

A detailed numerical solution algorithm for the current well-balanced UGKS is provided in Figure 1, and its implementation is available at the GitHub repository [21].

## 4. Numerical Experiments

In this section, we present numerical examples of a binary gas mixture to validate the well-balanced UGKS for multiscale and multicomponent flow. Multiscale simulations from free molecule flow to continuum two-species Euler solutions under a external force field are presented to demonstrate the capability of the unified scheme. The flow features in different regimes can be well captured by the unified scheme. Some interesting non-equilibrium phenomena, such as the characteristic behavior of different gas components in different flow regimes, are discussed. The hard sphere (HS) monatomic gas was employed in all test cases. With the overall number density $n=n_{1}+n_{2}$ and molecular diameter $d=(d_{1}+d_{2})/2$, the Knudsen number can be defined as

$$\mathrm{Kn}=\frac{1}{\sqrt{2}\pi d^{2}n},$$

and the parameter $\theta_{12}$ in Equation (7) becomes

$$\theta_{12}=\frac{4\sqrt{\pi }}{3}{\left(\frac{1}{\lambda_{1}}+\frac{1}{\lambda_{2}}\right)}^{1/2}\frac{1}{\sqrt{2}\pi \mathrm{Kn}\left(n_{1}+n_{2}\right)},$$

with which we can determine the modified macroscopic variables and collision frequency in Equations (5) and (6). The parameter β in Equation (6) was chosen to be the unit.

In the current calculations, we considered a binary gas mixture with γ=5/3 only. With the defined reference molecular mass and number density:

$$m_{ref}=\frac{m_{1}n_{1ref}+m_{2}n_{2ref}}{n_{1ref}+n_{2ref}},\rho_{0}=m_{ref}n_{ref}=m_{ref}\left(n_{1ref}+n_{2ref}\right),$$

the dimensionless variables are introduced as

$$\begin{array}{cc} \; & \hat{x}=\frac{x}{L_{0}},\hat{y}=\frac{y}{L_{0}},\hat{\rho }=\frac{\rho }{\rho_{0}},\hat{T}=\frac{T}{T_{0}},\\ \; & {\hat{u}}_{i}=\frac{u_{i}}{{\left(2kT_{0}/m_{ref}\right)}^{1/2}},{\hat{U}}_{i}=\frac{U_{i}}{{\left(2kT_{0}/m_{ref}\right)}^{1/2}},\hat{f}=\frac{f}{n_{0}{\left(2kT_{0}/m_{ref}\right)}^{3/2}},\\ \; & {\hat{P}}_{ij}=\frac{P_{ij}}{\rho_{0}\left(2kT_{0}/m_{ref}\right)},{\hat{q}}_{i}=\frac{q_{i}}{\left(\rho_{0}/2\right){\left(2kT_{0}/m_{ref}\right)}^{3/2}},{\hat{\phi }}_{i}=\frac{\phi_{i}}{2kT_{0}/\left(L_{0}m_{ref}\right)}, \end{array}$$

where $u_{i}$ is the particle velocity, $U_{i}$ is the macroscopic flow velocity, $P_{ij}$ is the stress tensor, $q_{i}$ is the heat flux, and $\phi_{i}$ is the external force acceleration. We drop the hat notation to denote dimensionless variables for simplicity henceforth.

### 4.1. Validation

In this part, we provide benchmark test cases to validate the current method. Both convection-dominated and diffusion-dominated flow problems are considered.

#### 4.1.1. Normal Shock Structure

The first case is the normal shock structure for a binary gas mixture [22]. The two components *A* and *B* are assumed to have a molecular diameter and different masses $m_{A}/m_{B}=2$. The upstream and downstream statuses are coupled by the Rankine–Hugoniot relationship, and the initial distribution functions are set as Maxwellian.

In the simulation, 100 uniform physical meshes were employed in physical domain x∈[−25,25] and 101 quadrature points were used in velocity space u∈[−10,10]. The upstream Mach number was Ma=1.5, and the Knudsen number was Kn=1.0. The CFL number was set to be 0.7. Different number density fractions $n_{A}/\left(n_{A}+n_{B}\right)=0.1$, 0.5, and 0.9 were considered.

**Figure 2:**
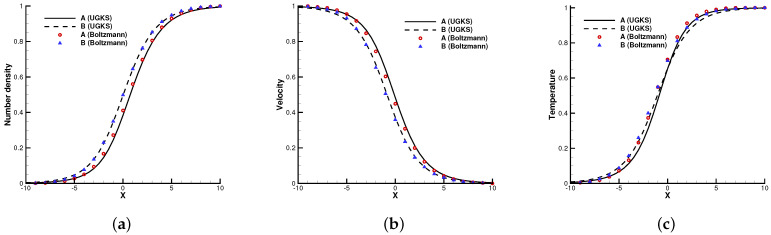
Normal shock profiles at $n_{A}/\left(n_{A}+n_{B}\right)=0.1$. (**a**) Number density. (**b**) Velocity. (**c**) Temperature.

**Figure 3:**
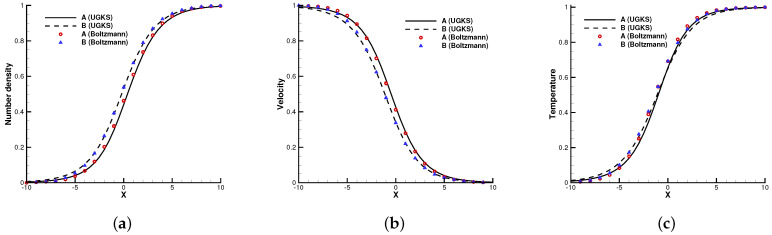
Normal shock profiles at $n_{A}/\left(n_{A}+n_{B}\right)=0.5$. (**a**) Number density. (**b**) Velocity. (**c**) Temperature.

**Figure 4:**
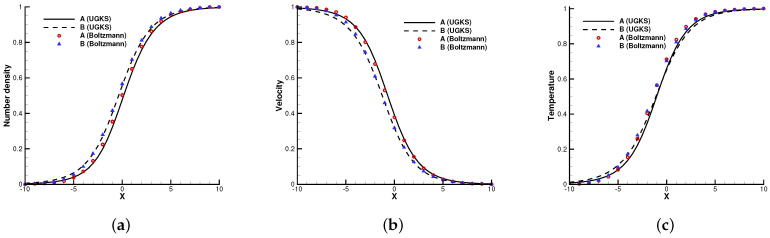
Normal shock profiles at $n_{A}/\left(n_{A}+n_{B}\right)=0.9$. (**a**) Number density. (**b**) Velocity. (**c**) Temperature.

Figure 2, Figure 3 and Figure 4 present the normalized numerical solutions under different density concentrations. The benchmark solutions from the full Boltzmann equation computed by the fast spectral method [23,24] are provided as a reference. As can be seen, the results from the UGKS and the Boltzmann equation exhibit good agreement under different number density fractions. This test case validates the UGKS in convection-dominated non-equilibrium flows.

#### 4.1.2. Fourier Flow

The second case is the Fourier flow. The two gas components were set in the same way as Section 4.1.1. The heat transfer problem was considered between two walls with different temperatures, i.e., $T_{L}=1$ and $T_{R}=0.5$. Maxwell’s diffusive boundary condition was considered at both ends. The initial gas was stationary and had a uniform density and temperature.

In the simulation, 100 uniform physical meshes were employed in physical domain x∈[0,1] and 72 quadrature points were used in velocity space u∈[−8,8]. The CFL number was set to be 0.7. Different Knudsen numbers were considered, i.e., Kn=1 and 0.1.

**Figure 5:**
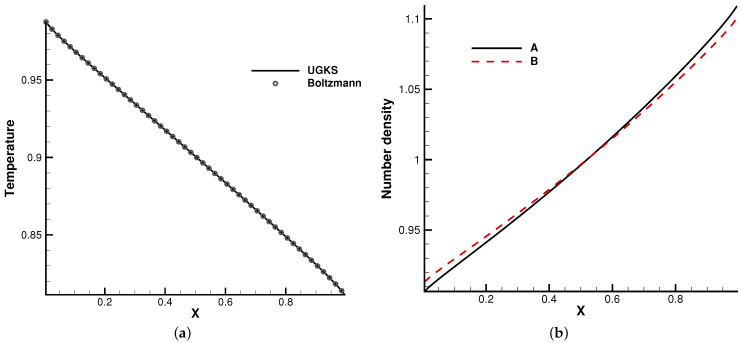
Kn=0.1. (**a**) Temperature. (**b**) Number density.

**Figure 6:**
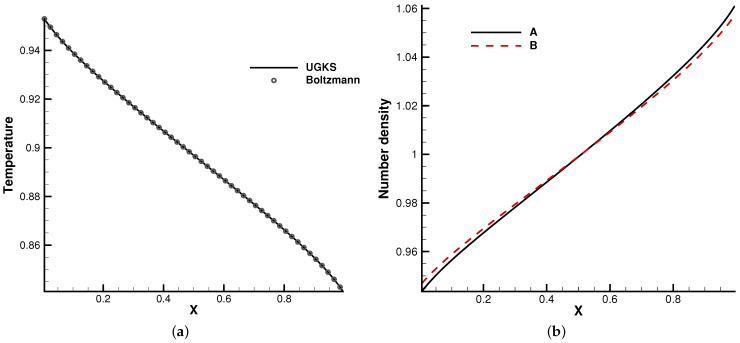
Kn=1. (**a**) Temperature. (**b**) Number density.

Figure 5 and Figure 6 present the temperature and density profiles. The benchmark full Boltzmann solutions are provided as a reference. It is clear that good agreement between the UGKS and reference solutions was achieved. In the rarefied regime, the number density profiles of two components deviate. Due to the different average molecular speeds, light molecules *B* tend to move towards hot regions, while heavy molecules to cold regions. This is a typical non-equilibrium flow phenomenon, which corresponds to the Soret effect [25]. In addition, the conservation of the system was checked. After 50 dimensionless time units when the convergent solution was obtained, the absolute error of the total mass was below 0.004‰. This test case validates the UGKS in diffusion-dominated non-equilibrium flows.

### 4.2. Perturbed Hydrostatic Equilibrium Solution

In the first test case, we studied the one-dimensional wave propagation from the hydrostatic equilibrium flow field [3]. The binary gas mixture was stillinitially at the hydrostatic equilibrium solution, and the domain x∈[0,1] was under the external force field $\phi_{x}=-1.0$, which points towards the negative *x*-direction, i.e.,

$$\rho_{0}\left(x\right)=p_{0}\left(x\right)=\exp\left(\phi_{x}x\right),u_{0}\left(x\right)=0.$$

The equilibrium solution was perturbed by the following pressure perturbation:

$$p\left(x,t=0\right)=p_{0}\left(x\right)+0.01\exp\left(-100{\left(x-0.5\right)}^{2}\right).$$

Here, $\rho_{0}$ and $p_{0}$ are the total density and pressure. In the gas mixture, the molecular mass ratio $m_{2}/m_{1}$ and number density ratio $n_{2}/n_{1}$ need to be specified to distribute the partition of density and pressure for each gas component.

**Figure 7:**
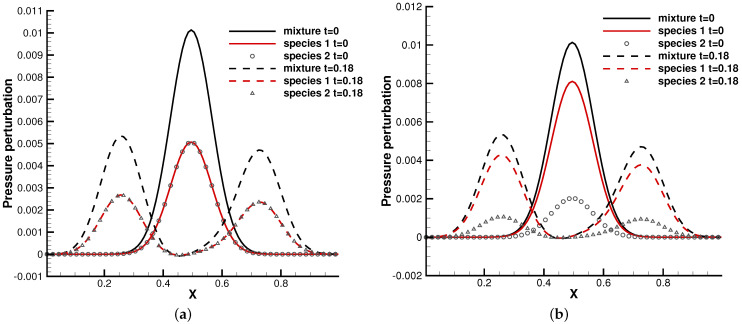
Pressure perturbation from a hydrostatic equilibrium solution. (**a**) $m_{2}/m_{1}=0.8$, $n_{2}/n_{1}=1$. (**b**) $m_{2}/m_{1}=0.5$, $n_{2}/n_{1}=0.25$.

In the simulation, 100 uniform physical meshes were employed in the physical domain and 101 quadrature points were used in the velocity space. The continuum flow regime was considered, and the Knudsen number was set as $10^{-5}$. Two cases were simulated with different molecular mass and number density ratios. The first case was set up with $m_{2}/m_{1}=0.8$, $n_{2}/n_{1}=1$, while in the second case, $m_{2}/m_{1}=0.5$, $n_{2}/n_{1}=0.25$. Figure 7 shows the pressure profiles at t=0.18 in the two cases. It can be seen that the small perturbation was well captured by the current well-balanced scheme without destroying the equilibrium solution in the bulk region. Such a capability is due to the unified treatment of particle transports and collisions under an external force field, as analyzed in [3,6]. Due to frequent intermolecular collisions in the continuum regime, different gas components behave coincidentally as a simple gas.

### 4.3. Riemann Problem under an External Force Field

Next, we considered the discontinuous solutions developed in the hyperbolic system. The Sod shock tube problem was employed as the test case [7]. Similarly, two cases were considered with different molecular mass and number density ratios. In the first case, it was set up with $m_{2}/m_{1}=0.5$, $n_{2}/n_{1}=1$, and $m_{2}/m_{1}=0.5$, $n_{2}/n_{1}=0.25$ in the second case. The initial condition was set as

ρ=1.0,U=0.0,p=1.0,x≤0.5,

ρ=0.125,U=0.0,p=0.1,x>0.5.

In the simulation, the external force $\phi_{x}=-1.0$ that points leftwards was considered. Different Knudsen numbers in the reference state were considered, Kn=0.0001, Kn=0.01, and Kn=1.0, with respect to different flow regimes. The computational domain x∈[0,1] was divided into 100 cells, and 101 quadrature points were used in the velocity space. The specular reflection boundary condition was employed at both ends.

**Figure 8:**
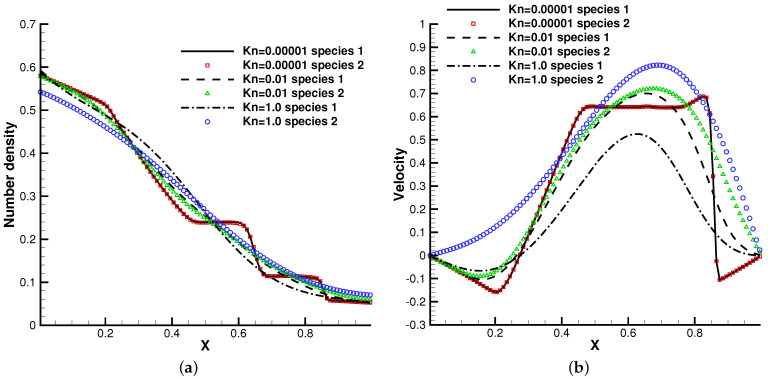

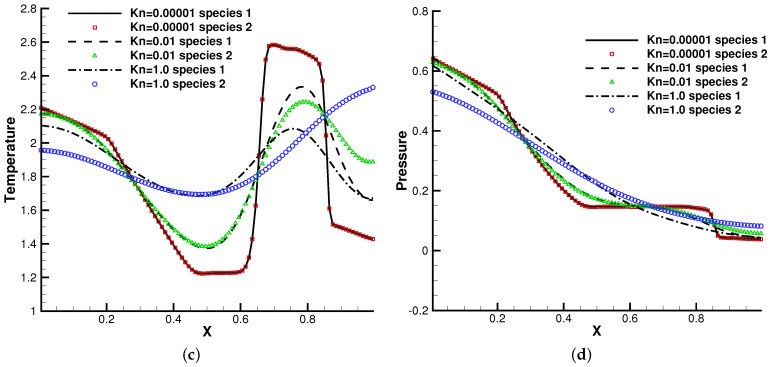
Sod test under an external force field with $m_{2}/m_{1}=0.5$, $n_{2}/n_{1}=1$. (**a**) Number density. (**b**) Velocity. (**c**) Temperature. (**d**) Pressure.

**Figure 9:**
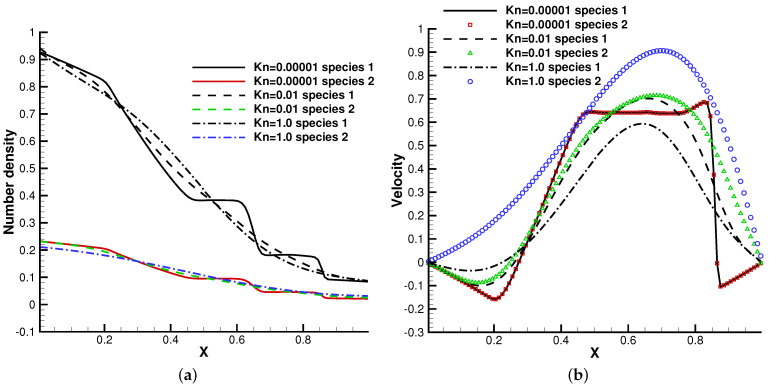

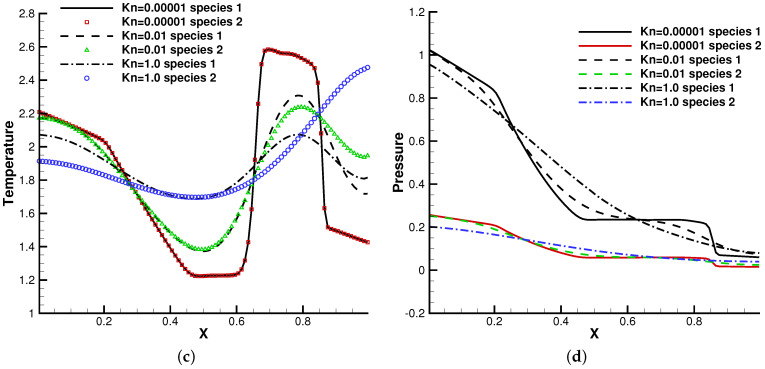
Sod test under an external force field with $m_{2}/m_{1}=0.5$, $n_{2}/n_{1}=0.25$. (**a**) Number density. (**b**) Velocity. (**c**) Temperature. (**d**) Pressure.

The profiles of macroscopic variables at t=0.2 are presented in Figure 8 and Figure 9. Under an external force field, the particles were driven towards the negative *x*-direction, resulting in the appearance of negative flow velocity near the left tube end. In comparison with the case without gravity, the thermodynamic quantities such as density, temperature, and pressure in the left side of the tube increase all together.

This numerical experiment validates the capability of the current method to simulate discontinuous cross-scale flow physics under an external force field. In the continuum limit with Kn=0.0001, the limited resolution in space and time results in the two-species Euler solution, and the current scheme plays the role of a shock-capturing algorithm. The frequent collisions prevent the particle penetration between fluid elements, and different gas components show consistent behaviors, just like a single gas. With the increment of the Knudsen number and the collision time, the degree of freedom for the free transport of individual gas components increases and the flow physics changes significantly. There is a smooth transition from the Euler solution of the Riemann problem to a collisionless Boltzmann solution. As different gas components have a specific molecular mass, the light gas transports much faster than the heavier one in the tube, which is shown in Figure 8b and Figure 9b.

### 4.4. Rayleigh–Taylor Instability

We turn to the two-dimensional case and consider the Rayleigh–Taylor instability [3]. The initial condition of the gas dynamic system in a polar coordinate (r,θ) was set as

$$\rho_{0}\left(r\right)=e^{-\alpha (r+r_{0})},p_{0}\left(r\right)=\frac{1.5}{\alpha }e^{-\alpha (r+r_{0})},U_{0}=0,$$

where

$$\left\{\begin{array}{c} \alpha =2.68,r_{0}=0.258,\;r\leq r_{1},\\ \alpha =5.53,r_{0}=-0.308,\;r>r_{1}, \end{array}\right.\mathrm{and}\left\{\begin{array}{c} r_{1}=0.6\left(1+0.02\cos\left(20\theta \right)\right),\;\mathrm{for}\;\mathrm{density},\\ r_{1}=0.62324965,\;\mathrm{for}\;\mathrm{pressure}. \end{array}\right.$$

The molecular mass and number density ratio in the gas mixture was set up with $m_{2}/m_{1}=0.8$, $n_{2}/n_{1}=1$, and $m_{2}/m_{1}=0.25$, $n_{2}/n_{1}=1$. The external force potential satisfies dΦ/dr=1.5, and the force points towards the origin of the polar coordinates. Different Knudsen numbers in the reference state were considered as Kn=0.0001, 0.01, and 1.0, The computational domain was divided into 60×60 uniform cells, and 29×29 quadrature points were used in the velocity space. The specular reflection condition was considered at all boundaries. Due to the density inversion contained in the initial flow field, the Rayleigh–Taylor instability will occur naturally as time evolves. A well-balanced method is expected to capture the flow motions around the unstable interface, while keeping the hydrostatic equilibrium solution in the bulk region.

**Figure 10:**
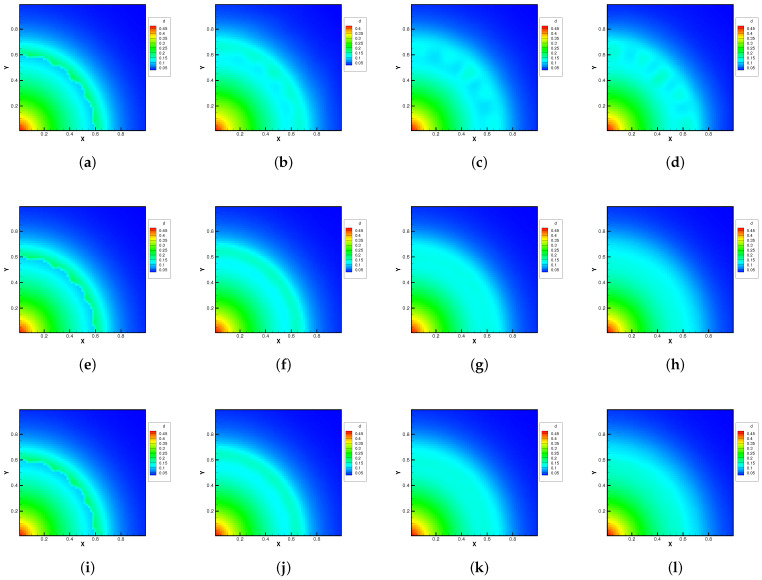
Density evolution under gravity at $m_{2}/m_{1}=0.8$ and reference Knudsen numbers 0.0001 (1st row), 0.01 (2nd row), and 1 (3rd row). (**a**) t = 0. (**b**) t = 0.8. (**c**) t = 1.4. (**d**) t = 2.0. (**e**) t = 0. (**f**) t = 0.08. (**g**) t = 0.16. (**h**) t = 0.24. (**i**) t = 0. (**j**) t = 0.08. (**k**) t = 0.16. (**l**) t = 0.24.

**Figure 11:**
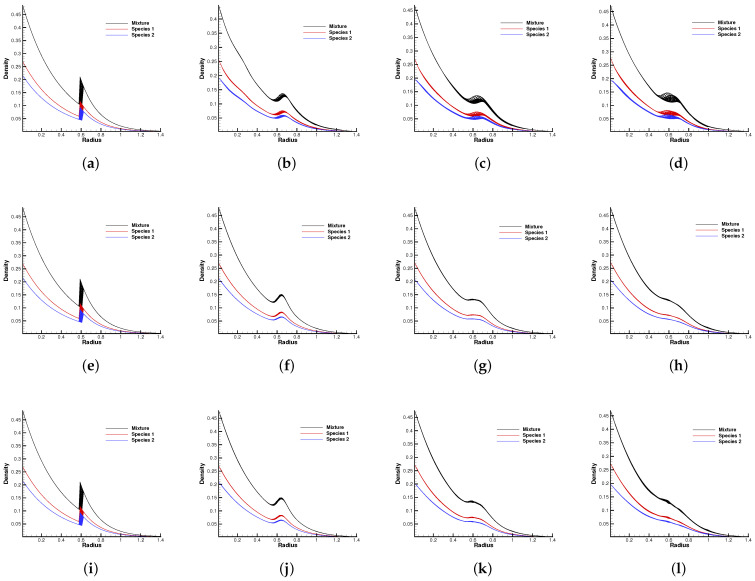
Density distribution along the radial direction at $m_{2}/m_{1}=0.8$ and reference Knudsen numbers 0.0001 (1st row), 0.01 (2nd row), and 1 (3rd row). (**a**) t = 0. (**b**) t = 0.8. (**c**) t = 1.4. (**d**) t = 2.0. (**e**) t = 0. (**f**) t = 0.08. (**g**) t = 0.16. (**h**) t = 0.24. (**i**) t = 0. (**j**) t = 0.08. (**k**) t = 0.16. (**l**) t = 0.24.

**Figure 12:**
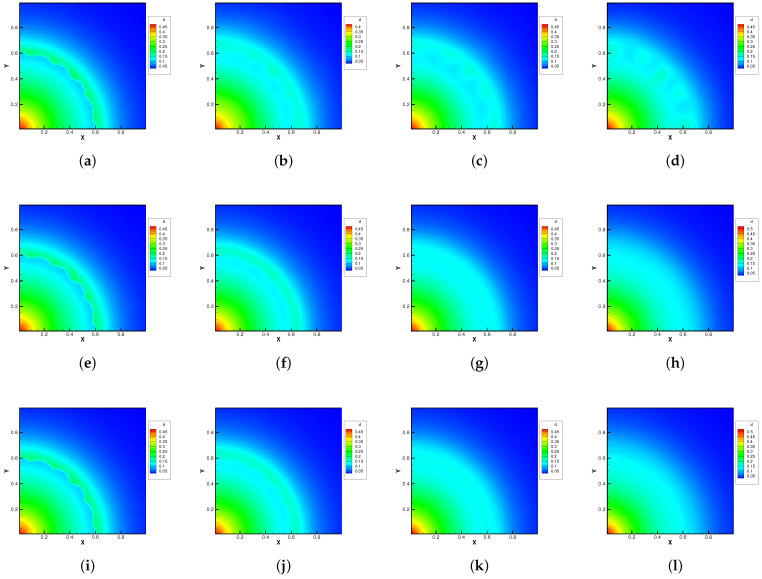
Density evolution under gravity at $m_{2}/m_{1}=0.25$ and reference Knudsen numbers 0.0001 (1st row), 0.01 (2nd row), and 1 (3rd row). (**a**) t = 0. (**b**) t = 0.8. (**c**) t = 1.4. (**d**) t = 2.0. (**e**) t = 0. (**f**) t = 0.08. (**g**) t = 0.16. (**h**) t = 0.24. (**i**) t = 0. (**j**) t = 0.08. (**k**) t = 0.16. (**l**) t = 0.24.

**Figure 13:**
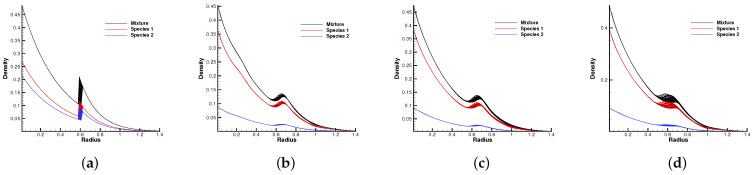

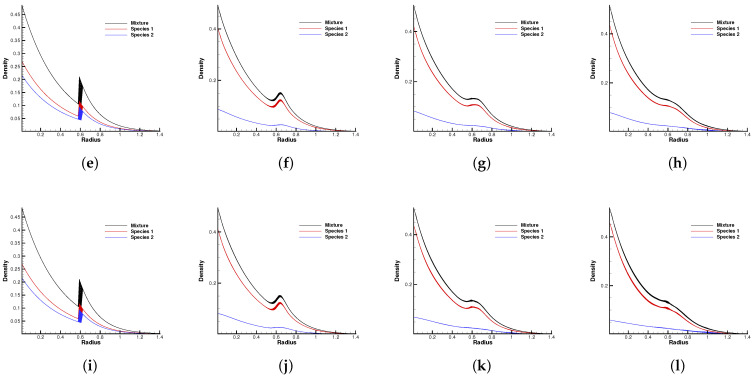
Density distribution along the radial direction at $m_{2}/m_{1}=0.25$ and reference Knudsen numbers 0.0001 (1st row), 0.01 (2nd row), and 1 (3rd row). (**a**) t = 0. (**b**) t = 0.8. (**c**) t = 1.4. (**d**) t = 2.0. (**e**) t = 0. (**f**) t = 0.08. (**g**) t = 0.16. (**h**) t = 0.24. (**i**) t = 0. (**j**) t = 0.08. (**k**) t = 0.16. (**l**) t = 0.24.

**Figure 14:**
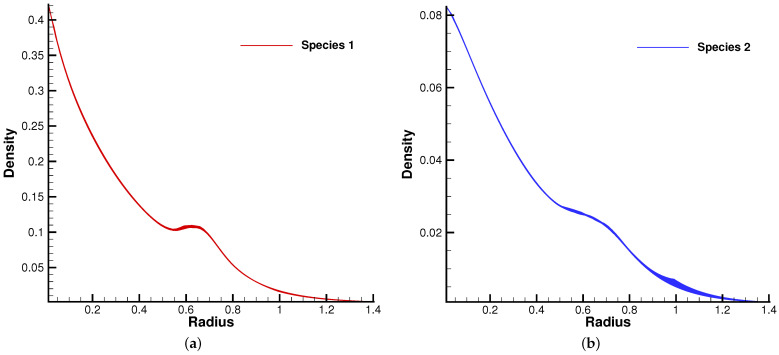
Density distribution for two gas components along the radial direction at t=0.08 with reference Knudsen number 0.01. (**a**) Species 1. (**b**) Species 2.

Figure 10 and Figure 11 plot the density contours and cross-sections of densities in all cells versus the radius with $m_{2}/m_{1}=0.8$ at different output times under different Knudsen numbers Figure 12 and Figure 13 present the same results with $m_{2}/m_{1}=0.25$. As can be seen, in different flow regimes, different flow physics emerge around the Rayleigh–Taylor interface. In the continuum regime, the frequent intermolecular interactions provide the effective mechanism to quickly initiate and strengthen the flow mixing. As the Kn increases, the particle transport phenomena dominate the flow evolution, and thus, the particles have a greater chance of penetrating directly through the mixing layer into the inner zone. Therefore, the strength of the Rayleigh–Taylor instability is greatly reduced. Due to the fact that different gas components have different molecular masses, the profiles of different species can be different, corresponding to different Knudsen numbers. Figure 14 presents the density profiles of the two components at t=0.08 and Kn=0.01. It is clear that, while the lighter components have already completed the density inversion, the heavy components are still in the mixing process. This is due to the fact that molecules with smaller masses have a faster mean speed. In all cases, it is clear that the hydrostatic solution is well preserved by the current well-balanced scheme, and the mixing of fluids occurs locally.

### 4.5. Lid-Driven Cavity under Gravity

The lid-driven cavity problem is a standard test case for both hydrodynamic and kinetic solvers, which contains complex flow physics related to compressibility, shearing structure, heat transfer, the boundary effect, non-equilibrium effects, etc. In this case, we calculated a lid-driven cavity problem under an external force, which serves as a typical case for the multiscale algorithms.

A binary gas mixture is enclosed by four walls with L=1. The upper wall moves in a tangential direction with a velocity $U_{w}=0.15$. The external force was set to be $\phi_{y}=0.0,-0.5,-1.0$, respectively, in the negative *y*-direction. The magnitude of gravity $\phi_{y}$ is denoted by *g*. The initial density and pressure were set up with

$$\rho \left(x,y,t=0\right)=2\exp\left(\phi_{y}y\right),p\left(x,y,t=0\right)=\exp\left(\phi_{y}y\right).$$

The molecular mass and number density ratio in the gas mixture was set up with $m_{2}/m_{1}=0.5$, $n_{2}/n_{1}=1$.

The Knudsen number in the reference state was set as Kn=0.05. There were 45×45 uniform cells used in the physical space and 41×41 quadrature points used in the velocity space. Maxwell’s diffusive boundary condition was used throughout the computation, and the wall temperature was $T_{w}=1$.

**Figure 15:**
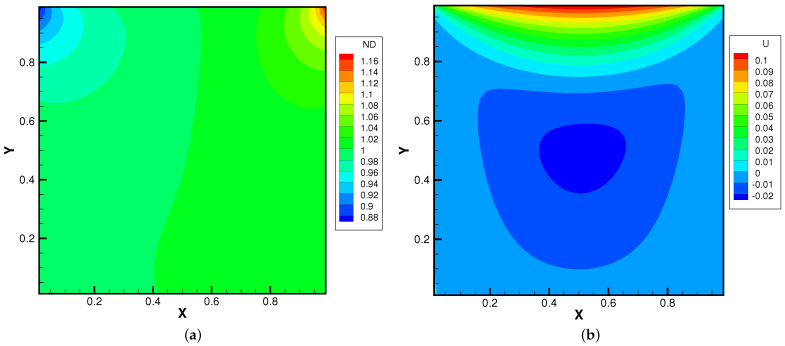

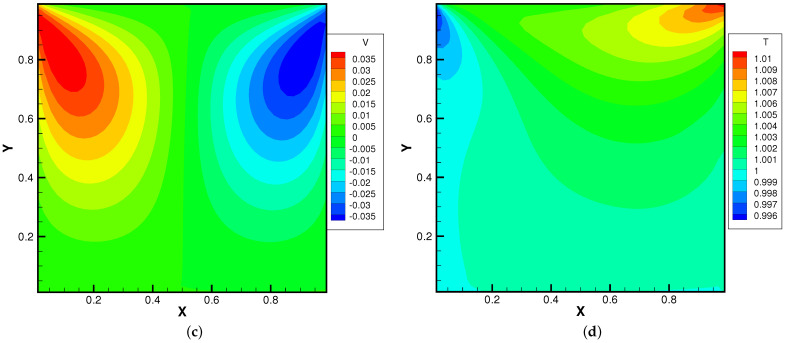
Lid-driven cavity solutions at $Kn_{ref}=0.05$ and $\phi_{y}=0$. (**a**) Number density. (**b**) U-velocity. (**c**) V-velocity. (**d**) Temperature.

**Figure 16:**
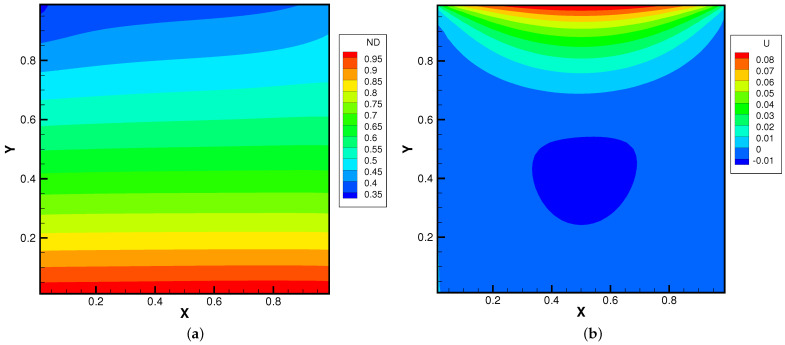

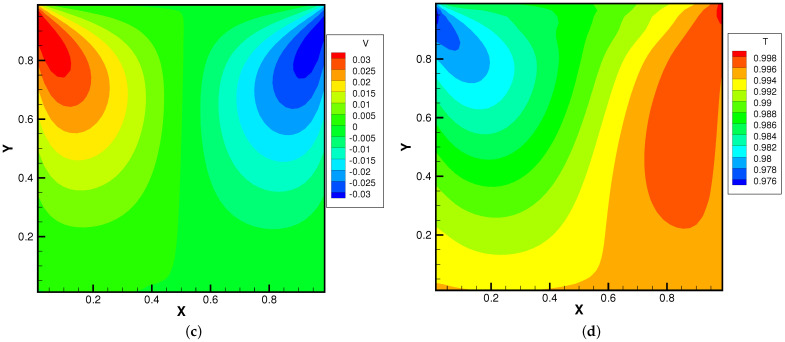
Lid-driven cavity solutions at $Kn_{ref}=0.05$ and $\phi_{y}=-0.5$. (**a**) Number density. (**b**) U-velocity. (**c**) V-velocity. (**d**) Temperature.

**Figure 17:**
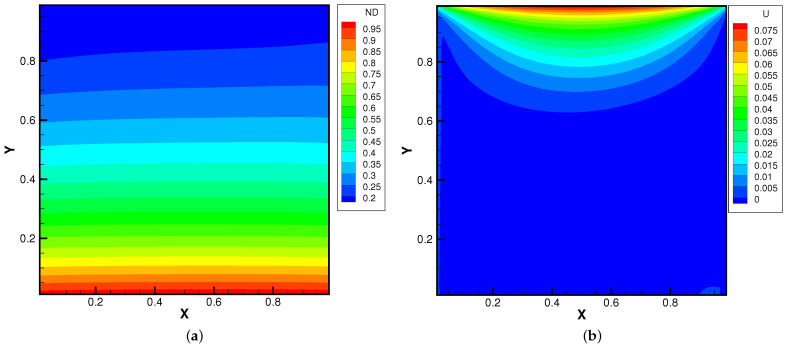

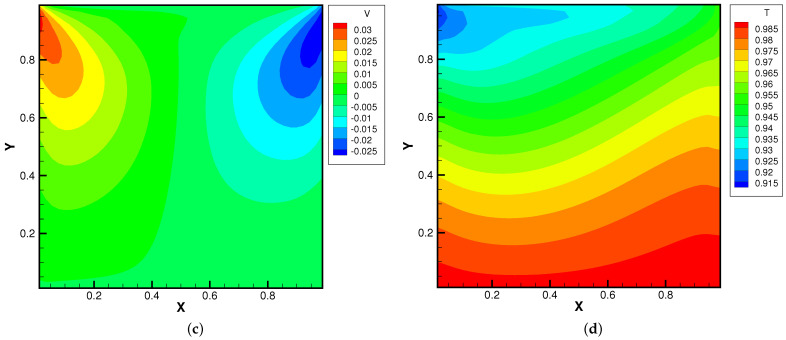
Lid-driven cavity solutions at $Kn_{ref}=0.05$ and $\phi_{y}=-1.0$. (**a**) Number density. (**b**) U-velocity. (**c**) V-velocity. (**d**) Temperature.

**Figure 18:**
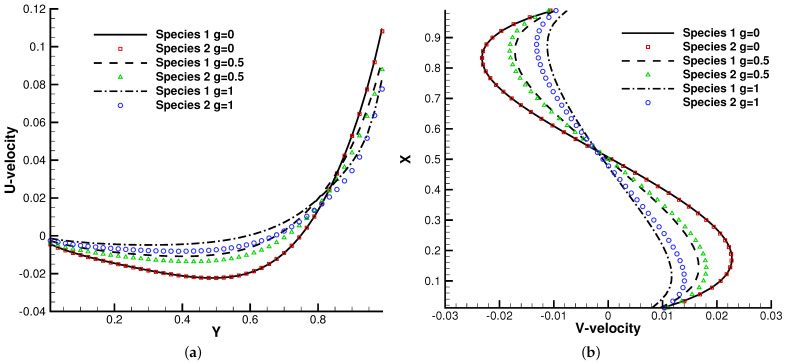
U,V velocity along the horizontal and vertical center lines of the cavity. (**a**) U-velocity. (**b**) V-velocity.

**Figure 19:**
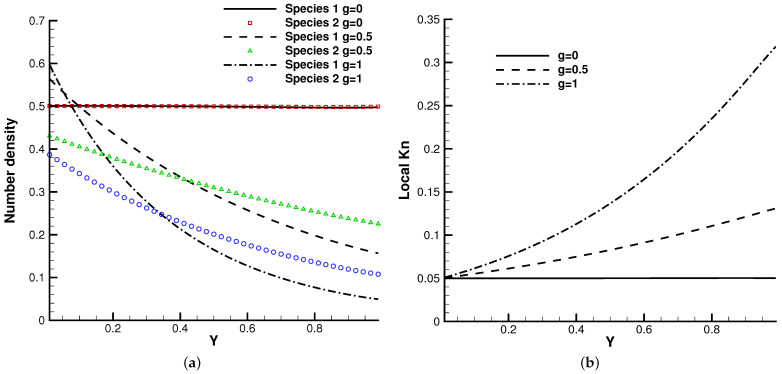
Number density and local Knudsen number along the vertical center line of the cavity. (**a**) Number density. (**b**) Local Knudsen number.

Figure 15, Figure 16 and Figure 17 present the numerical solutions related to different magnitudes of the external force. Due to the existence of a force field, along the forcing direction, the gas density changes significantly along the vertical direction of the cavity, as does the local Knudsen number. As as result, the gas inside the cavity, depending on the position of the *y*-axis, can stay in different flow regimes. Similar to the results of a single-component gas [26], the temperature of the gas around the upper surface of the cavity decreases in spite of the viscous heating effect. Such a phenomenon happens during the energy exchange among gravitational and kinetic energy and can be explained as a result of the non-equilibrium heat transfer driven by an external force. Different from the equilibrium thermodynamics, the shift and distortion of the gas distribution function due to the external forcing term provide the dominant mechanism for particle transports, especially in the rarefied regions. The density and velocity distributions at the central lines of the cavity, as well as the local Knudsen number are presented in Figure 18 and Figure 19. As plotted, the increased external force results in the stabilizing effect, i.e., to reduce the rotating speed of the main vortex. With the increment of the force magnitude, the velocity profile is flattened, indicating a weaker vortex motion. This numerical results validates the current well-balanced method for the study of non-equilibrium flows under an external force field.

## 5. Conclusions

The atmosphere is composed of multicomponent flows under an external force. In this paper, a well-balanced unified gas-kinetic scheme for multicomponent flows has been developed. The well-balanced property of the unified scheme was validated through both theoretical demonstrations and numerical tests. The detailed strategy for the construction of the current algorithm was illustrated. Many numerical cases were provided to validate the scheme. New physical observations, such as the consistent transport in the hydrodynamic regime and the decoupled transport in the rarefied regime of different components, were clearly identified and discussed. The well-balanced UGKS provides an alternative choice for the study of real non-equilibrium gaseous flow on the Earth and beyond, which is useful in astronautical and astrophysical applications.

## Data Availability

The research data are available at https://github.com/vavrines/UGKS (11 August 2022).

## References

[1] Abgrall R. (1996). How to Prevent Pressure Oscillations in Multicomponent Flow Calculations: A Quasi Conservative Approach. J. Comput. Phys..

[2] Fedkiw R.P., Aslam T., Merriman B., Osher S. (1999). A Non-oscillatory Eulerian Approach to Interfaces in Multimaterial Flows (the Ghost Fluid Method). J. Comput. Phys..

[3] LeVeque R.J., Bale D.S. (1999). Wave propagation methods for conservation laws with source terms. Hyperbolic Problems: Theory, Numerics, Applications.

[4] Botta N., Klein R., Langenberg S., Lützenkirchen S. (2004). Well balanced finite volume methods for nearly hydrostatic flows. J. Comput. Phys..

[5] Xing Y., Shu C.W. (2013). High order well-balanced WENO scheme for the gas dynamics equations under gravitational fields. J. Sci. Comput..

[6] Tian C., Xu K., Chan K., Deng L. (2007). A three-dimensional multidimensional gas-kinetic scheme for the Navier–Stokes equations under gravitational fields. J. Comput. Phys..

[7] Luo J., Xu K., Liu N. (2011). A well-balanced symplecticity-preserving gas-kinetic scheme for hydrodynamic equations under gravitational field. SIAM J. Sci. Comput..

[8] Chen S., Guo Z., Xu K. (2020). A Well-Balanced Gas Kinetic Scheme for Navier–Stokes Equations with Gravitational Potential. Commun. Comput. Phys..

[9] Xu K., Huang J.C. (2010). A unified gas-kinetic scheme for continuum and rarefied flows. J. Comput. Phys..

[10] Xu K. (2015). Direct Modeling for Computational Fluid Dynamics: Construction and Application of Unified Gas-Kinetic Schemes.

[11] Xiao T., Cai Q., Xu K. (2017). A well-balanced unified gas-kinetic scheme for multiscale flow transport under gravitational field. J. Comput. Phys..

[12] Prestininzi P., La Rocca M., Montessori A., Sciortino G. (2014). A gas-kinetic model for 2D transcritical shallow water flows propagating over dry bed. Comput. Math. Appl..

[13] Schotthöfer S., Xiao T., Frank M., Hauck C.D. Structure Preserving Neural Networks: A Case Study in the Entropy Closure of the Boltzmann Equation. Proceedings of the International Conference on Machine Learning, PMLR.

[14] Xiao T., Frank M. (2021). A stochastic kinetic scheme for multi-scale plasma transport with uncertainty quantification. J. Comput. Phys..

[15] Bhatnagar P.L., Gross E.P., Krook M. (1954). A model for collision processes in gases. I. Small amplitude processes in charged and neutral one-component systems. Phys. Rev..

[16] Andries P., Aoki K., Perthame B. (2002). A consistent BGK-type model for gas mixtures. J. Stat. Phys..

[17] Morse T. (1963). Energy and momentum exchange between nonequipartition gases. Phys. Fluids.

[18] Bird R.B. (2002). Transport phenomena. Appl. Mech. Rev..

[19] Slyz A., Prendergast K.H. (1999). Time-independent gravitational fields in the BGK scheme for hydrodynamics. Astron. Astrophys. Suppl. Ser..

[20] Xiao T., Xu K., Cai Q., Qian T. (2018). An investigation of non-equilibrium heat transport in a gas system under an external force field. Int. J. Heat Mass Transf..

[21] Xiao T. (2021). Kinetic. jl: A portable finite volume toolbox for scientific and neural computing. J. Open Source Softw..

[22] Kosuge S., Aoki K., Takata S. (2001). Shock-wave structure for a binary gas mixture: Finite-difference analysis of the Boltzmann equation for hard-sphere molecules. Eur. J. Mech.-B/Fluids.

[23] Wu L., White C., Scanlon T.J., Reese J.M., Zhang Y. (2013). Deterministic numerical solutions of the Boltzmann equation using the fast spectral method. J. Comput. Phys..

[24] Xiao T., Xu K., Cai Q. (2019). A unified gas-kinetic scheme for multiscale and multicomponent flow transport. Appl. Math. Mech..

[25] Rahman M., Saghir M. (2014). Thermodiffusion or Soret effect: Historical review. Int. J. Heat Mass Transf..

[26] Xiao T. (2021). A flux reconstruction kinetic scheme for the Boltzmann equation. J. Comput. Phys..

